# International students’ experience of a western medical school: a mixed methods study exploring the early years in the context of cultural and social adjustment compared to students from the host country

**DOI:** 10.1186/s12909-015-0394-2

**Published:** 2015-07-02

**Authors:** A. McGarvey, R. Brugha, R. M. Conroy, E. Clarke, E. Byrne

**Affiliations:** 1 Anatomy Department, Royal College of Surgeons in Ireland, 123 St Stephen’s Green, Dublin 2, Ireland; 2 Division of Population and Health Sciences, Royal College of Surgeons in Ireland, 123 St Stephen’s Green, Dublin 2, Ireland; 3 Health Professions Education Centre, Royal College of Surgeons in Ireland, 123 St Stephen’s Green, Dublin 2, Ireland

**Keywords:** International, Students, Culture, Medical education, Acculturation, Internationalisation, Social adjustment

## Abstract

**Background:**

Few studies have addressed the challenges associated with international students as they adapt to studying medicine in a new host country. Higher level institutions have increasing numbers of international students commencing programmes. This paper explores the experiences of a cohort of students in the early years of medical school in Ireland, where a considerable cohort are from an international background.

**Methods:**

A mixed exploratory sequential study design was carried out with medical students in the preclinical component of a five year undergraduate programme. Data for the qualitative phase was collected through 29 semi-structured interviews using the peer interview method. Thematic analysis from this phase was incorporated to develop an online questionnaire combined with components of the Student Adaptation to College Questionnaire and Student Integration Questionnaire. First year students were anonymously surveyed online. The Mokken Scaling procedure was used to investigate the students’ experiences, both positive and negative.

**Results:**

Three main themes are identified; social adjustment, social alienation and cultural alienation. The response rate for the survey was 49 % (467 Respondents). The Mokken Scaling method identified the following scales (i) Positive experience of student life; (ii) Social alienation, which comprised of negative items about feeling lonely, not fitting in, being homesick and (iii) Cultural alienation, which included the items of being uncomfortable around cultural norms of dress and contact between the sexes. With the threshold set to *H* = 0.4. Subscales of the positive experiences of student life scale are explored further.

**Conclusions:**

Overall student adjustment to a western third level college was good. Students from regions where cultural distance is greatest reported more difficulties in adjusting. Students from these regions also demonstrate very good adaptation. Some students from the host country and more similar cultural backgrounds were also struggling. Acculturation is more complex than being associated with cultural distance and worthy of further exploration.

## Background

Global student mobility is a rapidly growing phenomenon that has had a major impact on the management of students and institutional resources across the world [1–3]. The number of students travelling to another country for higher education has increased by 65 % since 2000 amounting to 3.3 million students globally [1–3].

Acculturation is the process whereby change takes place as a result of two or more cultures coming into contact [4]. Acculturation occurs among international students as well as with the host culture. It is acknowledged that moving to a foreign country can bring a number of challenges and international students may experience stress and adjustment issues from life changes in the acculturation process [5].

Berry describes a stress and coping framework to explain factors that affect acculturative stress and adaptation and describes psychological acculturation as a significant life changing event [5, 6]. These changes are assessed by the individual and may be perceived as benign, or as opportunities, and hence not a stressor; or they can be viewed as difficulties and hence as acculturation stressors. These stressors may be low for the individual if they have appropriate coping strategies; if the coping strategies are not sufficient then the acculturative stress may be higher and potentially could present as depression or anxiety. Individuals who share a cultural heritage or who are settled in a common society do not necessarily experience or navigate or adapt to acculturation in the same way [5].

A significant body of research indicates that international students encounter life changes as a result of undertaking their education in a different culture. The changes can become stressors or hassles as termed by Safdar et al. [7], if they are described by the students as being a difficulty. A large number of studies have looked at student retention in higher education. Tinto [8] in particular addresses the need for students to participate in student culture, both inside the learning environment and outside in order to integrate socially. Baker and Sirkyk [9, 10] have also identified social and academic integration to have a positive influence on student performance. They identify four concepts in academic integration: academic, social, personal, and emotional adjustment and attachment. Social adjustment describes how students manage sociocultural demands, such as making friends. Personal and emotional adjustment looks at psychological and physical distress while adapting to the new academic challenges or lifestyle. Attachment looks at the affiliation to institutional goals.

More recent research indicates that social networks of students have a large influence on how first year students adapt to their new environment [1, 3, 11–13]. Russell at et al. [1, 14] conducted a large study on 979 international students and reported that 41 % of those students reported stress either as homesickness, cultural shock or perceived discrimination. Domestic students have been described as needing to focus on social integration less due to the proximity to family and home and indeed habituation to cultural norms [1, 15]. In light of these studies Rienties et al. [3] have added to the Baker and Siryk concepts by adding five further social integration factors that are targeted specifically at the international student namely; perception of faculty, social support by family and friends, social life, ethnic background and financial support.

In one of the very few papers looking at internationalisation in the Irish higher level context Dunne [15] investigated the host student experience of international students. He found that the host student differentiates themselves from international students from a national cultural perspective such as language, but also on their overall approach to the higher education experience. Levels of anxiety amongst the host students were identified as reasons to deter them from engaging with international students, perceiving a need to adapt their communication styles, leaving homophilic tendencies to simply override attempts to encourage voluntary intercultural interaction. Homophily is described as “the principle that contact between similar people occurs at a higher rate than among dissimilar people” [16]. It is clear that in order to understand the international student experience the host students’ experiences must also be taken into account as a comparator and a potential influencer of the student experience.

The literature to date looks at academic and social integration. However, very little work has been published addressing the experience of acculturation in higher education amongst medical students in particular. Here the dynamic is two-fold in that the students must first learn to adapt to a new host country and higher education. Internationalisation of medical education has been widely reviewed in the context of clinical electives [17]. The student must also acculturate to the host country’s healthcare environment. It is essential that medical educators remain attuned to their diverse students’ needs, engaging with their academic environment by adopting appropriate techniques and supports [2].

Previous research in this College, where the research reported on in this paper was conducted, found that medical students have similar integration issues to those of other international students [18]. Preliminary focus group studies revealed a number of concerns raised by the students from different cultural backgrounds. These included: alcohol consumption, challenges interacting with the opposite sex, independence, the ‘real’ Ireland as opposed to the perceived, traditional practices, perceptions of failing or poor academic attainment, and challenges with English as a second language. There is a need to further understand the experiences of the medical student as they adapt academically and socially within and outside of the higher education institution. Pre-clinical and clinical experiences are likely to cause different adaptation and integration challenges. This study explores the experiences of international students during their early years in the context of cultural and social adjustments.

A number of previous studies investigating international student cohorts have consisted of students mainly from one other continent, for example in Australian studies where the international student body is predominantly Asian in origin [1, 2]. The medical school in which the present study has been undertaken accommodates students with a greatly diverse international representation and is therefore well placed to look at the student experience and reactions from many parts of the world using the host students as a comparator. It reports on the challenges faced by these students culturally and socially as they adapt to their new surroundings.

**Fig. 1:**
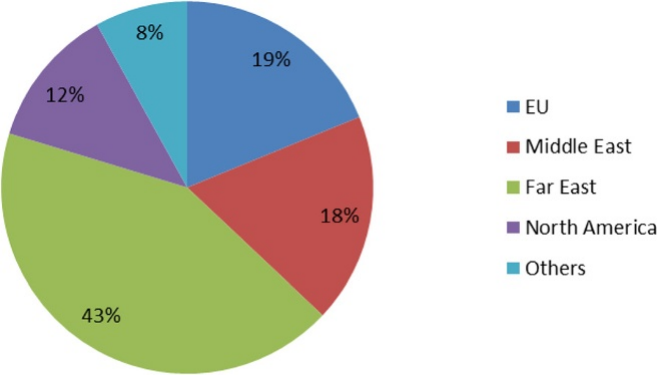
Representation of RCSI years 1 & 2 medical students based on region of origin for academic year 2014 / 2015

The regional make-up of the medical student body of the medical school where this study was undertaken in the first two years of the direct entry (five year) programme is shown in Fig. 1. A number of activities take place within this higher education institution to encourage social engagement and intercultural interaction. Initial induction programmes occur during week 1 of the programme in year 1. In addition, when students are divided into small groups for projects and teaching purposes, groups are purposely formed to have a cultural mix in place.

## Methods

The data reported herein is part of a mixed methods exploratory sequential study design [19]. A preliminary document review of previous focus group discussions conducted in the college [18], internal documents, such as policies, reports, web sites where available and activities relating to cultural diversity were reviewed before the study commenced. The study was approved by the RCSI Research Ethics Committee (Reference number: REC566bb).

### Data collection (phase 1)

Sixteen Year 2 medical undergraduates were recruited to conduct 31 peer interviews with Year 1 students. A shared cultural identity or commonality between interviewer and interviewee can be advantageous in carrying out in-depth interviews [20] and to that end an effort was made, in so far as possible, to match interviewer and interviewee for gender and cultural background. Five broad geographic/cultural categories, representing the student body makeup were identified – Ireland and Europe, Canada and US, Africa and Caribbean, Middle East and Asia. Diversity of students was sought not to claim generalisability of findings across particular student groups, but to obtain as diverse experiences as possible. The students as mentioned were from diverse backgrounds and had often spent much of their life living or studying in countries other than their home country (Table 1).

**Table 1:** Final profile of year 2 student interviewers (*N* = 16)

	Ireland and EU	Canada and US	Africa and Caribbean	Middle East	Malaysia and Asia	Total
MEN	1	0	1	2	1	5
WOMEN	2	3	1	3	2	11
Total	3	3	2	5	3	16

Year 1 students in their first semester were recruited as interviewees. A detailed information sheet and copy of the consent form were forwarded to the interested students. The final number of Year 1 volunteers was 31 (Table 2). Both interviewers and interviewees were recruited following presentations to the classes outlining the project, the process of peer interviewing and their specific roles. Volunteer interviewers (*n* = 16) attended one or two half-day training workshops.

**Table 2:** Profile of year 1 student interviewees (*N* = 31)

	Ireland and EU	EU	Canada and US	Africa and Caribbean	Middle East	Malaysia and Asia	Other	Total
MEN	2	0	2	0	3	2	0	9
WOMEN	1	1	6	2	5	6	1	22
Total	3	1	8	2	8	8	1	31

Semi-structured student interviews were completed in the college, a familiar place to all those involved, in Month 3 of their programme. Questions were devised based on previous work as described above [18] with input from the peer led interviewers. Interviewer/interviewee students were paired by one of the researchers based on gender and self-declared cultural background/country of origin and every effort was made to achieve as appropriate a match as possible. While exact cultural matching was not possible in all cases, all interview pairs were matched for gender. A total of 29 recorded interviews were obtained over the two-week period. All Year 1 students were given the option of reviewing/amending the transcripts of their interviews, an option of which four students availed. Consent forms were signed by the interviewees both prior to and following the interview.

Three focus group discussions were also held to explore some of the issues raised in the interviews. One Middle Eastern and 2 mixed nationality focus group discussions were conducted.

### Data collection (phase 2)

For phase 2 of the study, an online survey was conducted twelve months after the initial interviews. This 81 item survey comprised three elements as outlined below (i – iii):

(i) Student adaptation to College questionnaire
  The student adaptation to college questionnaire (SACQ) [9] is composed of 62 items and divided in to four scales; academic adjustment, social adjustment, personal-emotional adjustment and attachment. For the purposes of this study only social adjustment and attachment were included. The remaining categories were not included as the focus was on student adaptation to a new social context.
(ii) Social integration questionnaire
  Student social integration was measured using the Social Integration Questionnaire (SIQ) [3]. This tool consists of 15 items divided into four constructs; perception of faculty, study support, students’ satisfaction with social life, and financial support.
(iii) Phase 1 qualitative findings
  Based on thematic analysis from the peer interviews 35 additional questions were included to assess the representativeness of the qualitative findings that were not covered by the other questions. The additional items addressed demographic issues, language spoken amongst family and type of second level school attended, identified ethnicity, preparation for moving to Ireland, usefulness of support systems developed within the college for induction purposes, homesickness, attitudes toward alcohol, and experience of discrimination.

The questionnaire was piloted with the assistance of senior medical students. Minor changes were then made to the survey prior to launch. Students from the first year and second year of the programme were invited to participate in the survey. The Student Union supported the study and an online video was made where staff and students encouraged student participation. The survey was run over a two week period with three invitations from one of the research team to the students to complete the survey using Survey Monkey. Anonymity was guaranteed.

### Data analysis

#### Phase 1

The individual interviews were transcribed and coded in NVivo 10 initially using the main headings from the interview topic guide. The first 10 interviews were coded separately by two members of the team and then reviewed to revise the coding schema. A number of inductive codes were then added to the previous themes derived from the interview topic guide. All the interviews were then coded using the revised coding scheme.

#### Phase 2

The questionnaire included groups of items written to tap different aspects of the student experience. A Mokken scaling exercise was performed on the items. Mokken scaling begins by selecting the two items with the greatest consistency – the fewest Guttman errors. It then adds further items, minimising the Guttman errors until no further items can be added without bringing the consistency of the whole scale below a specified threshold. In scale construction, the minimum threshold is usually set at a Loevinger H value of 0.3. The H coefficient is a measure of the amount of Gutman errors in the scale compared with the amount that would be expected by chance. A H of one means that there are no Guttman errors, while a score of zero means that the errors are equal to what you would expect by chance. When the Mokken procedure has created a scale, it then examines the item pool to see if further scales can be constructed with the remaining items. It halts when there are no more items that seem to scale together [21, 22].

Scale scores were calculated for the scales identified by the Mokken procedure. Differences between groups on these scale scores were examined using OLS regression with robust standard errors. Irish students were used as a baseline with other student groups entered as dummy terms into the regression model. Where scale scores were markedly skewed, Poisson regression with robust standard errors was substituted for OLS regression [23]. The distributions of individual scale scores are shown as strip plots with aligned boxplots showing summary quantiles for each group.

## Results

To facilitate ease of understanding the results from phase 1 and 2 are presented separately using the categories

1. Social adjustment
2. Social alienation
3. Cultural alienation

Other areas of interest will also be presented in the quantitative findings

### Phase 1

As mentioned above 29 interviews were recorded, transcribed and thematically analysed.

### Social adjustment

#### Independent living

Though it was common that students had some problems adjusting to a new life in Ireland, for a number of students the problems were minimal or non-existent. Much of this can be attributed to having lived away from home before or having family visit or living close by.

*I lived in America for a long time so coming to Ireland wasn’t such a huge shock. It was different because I spent my teenage life in XXXX and it’s very conservative and segregated and the whole lifestyle is different. I liked it when I came to Ireland. I liked it a lot because it reminded me of when I was a kid (JC29)*

However, what was more common was that many students found it difficult to balance their time between studying, their social life and managing their household affairs. This applied especially to those students who have moved away from home for the first time.

*In hindsight it seems pretty minor but at the time, I’d never done my own laundry, bought my own groceries, cooked my own food before moving over here and I think those three things were a kind of a hit in the face. Doing your own laundry, it took a while to find a schedule to always have clean clothes and you know, buying groceries. There are times when you go to the fridge and it’s empty and it sucks. There’s no worse feeling than that. So it was like, being on time about getting groceries, making sure you had food in the house and clean clothes. That was probably the thing that I stumbled up on the most (JC21).*

#### Homesickness, loneliness and depression

Most of the students interviewed missed home at some stage of their time in Ireland. This was either when they arrived first or on particular occasions, such as when they were sick, eating meals or during exams. For some the level of homesickness decreased overtime, but for others the longer they stayed the more homesick they got.

*Well I guess every day I think of them sometimes. Especially when I’m eating or cooking with my friends. I want to eat my mum’s food and eat with my brother.... It’s better but it’s still kind of upper. .... I just keep thinking about them and sometimes I just can’t concentrate (on study). .... I don’t think I’ve overcome it. I’m kind of stuck (JC19).*

However, quite a few students did not feel homesick at all

*For most of the time since I was 16 each time I’m back home, it’s for a holiday. I tend to associate home with holiday and relaxing so whenever I’m stressed I do miss home. … When I left home when I was 16 I wasn’t homesick at all. I was just so excited to be leaving the country (JC23)*

Many students felt very lonely, not just homesick. Against their own expectations, some even become quite depressed.

*It was very hard. I’m the eldest of my siblings so I’m very used to taking care of myself and other people but still, being alone, not knowing who to be friends with, I was on survival mode. … What I struggled with most was emotional, being with friends, it’s really bad when things take you away from your study. They’re not supposed to but study shouldn’t take all of your life either. There has to be a balance (JC29).*

#### Making friends

Making friends was seen as one of the main factors affecting settling in. Most of the young adults interviewed had left behind friendships and social networks built up over many years and the task of making new friends was daunting. Some of the international students felt that it was easier to make friends with other international students as there was a common bond in transitioning. This group felt that the need they had for more intense relationships was something Irish people wouldn't understand as they had their established social networks which they did not have to leave behind.

*Just the friends you make here, they understand what you’re going through. A lot of them are foreign students. They’re also struggling, don’t know anyone or know what’s going on at all. So the friendships, the intimacy or closeness is just so much stronger than I would ever have experienced before. … The Irish, they just think you’re crazy. You want to plan or hang out and they have family and school friends, their whole life, they’re like what’s the big deal? They have a full life and your life is so empty (JC8)*

For some they hadn’t expected how long it would take to make close friends.

*Well it’s been a long time since I had to make new friends, I’ve been at home for so long. I think that was a bit intimidating and figuring out how to make friends and starting over. And also being a bit older in my year, it’s taken some time …(JC6)*

### Social alienation

#### Freedom of expression and friendliness

In general, students found Irish people to be friendly and welcoming. However some students expressed suspicion of this in the beginning.

*…so you come over here and everyone’s really nice and you’re like what, what is this business? (JC9)*

Students also felt that Ireland was open and accepting of differences.

*I’ve noticed that in Ireland people are more open to differences, they’re ok. They’re very, very friendly. .... Ireland is really open. I think if you asked this question in another European country people would be like, yes and no. Ireland is really open to a lot of different cultures. You see a lot of Spanish and French people here. I think they’re very open about it (JC29).*

For some there was the freedom of airing opinions and views without fearing repercussions from what they said.

*Even though in XXX it’s a democratic country, it’s a social thing. You don’t want people to look at you. Here it’s like, especially when things that are taboo in XXX they’re acceptable here. You wouldn’t be bothered mentioning it and you wouldn’t be worried about what they might think of you. Are they going to tell it to another person? You don’t have to think about that so there are no restrictions (JC13).*

### Cultural alienation

#### Personal space

Culturally, personal space is viewed differently. There was significant discomfort, and an awareness of the discomfort caused to others, in terms of acceptable closeness, such as how close to sit to another person, or physical contact with another person, such as hugging, shaking hands and kissing. Underlying the cultural implications of personal space were gender and religious dimensions– notably, issues with closeness of physical contact between sexes amongst Islamic students.

*The only thing that used to make me feel uncomfortable is the fact that people will sometimes just invade your personal space. You’re like no, just step a little bit further. …I remember I was talking to someone and this guy wanted to pass, he didn’t even ask me to move and he just passed and he was too close to me and I was like, don’t get too close. I didn’t say anything. You just get used to it. (It wouldn't happen in XXX… You’d get slapped. If a guy did that he’d get slapped (JC13).*

Many of the students were aware of differing practices among students regarding hugging or shaking hands. Students were aware that by not accepting a hand shake they might be viewed as discourteous or rude and so for many they shook hands when needed. Others reported simply forgetting at times that their friend would not be comfortable shaking their hands or not that welcoming of a birthday hug.

*When I first came here and guys want to shake hands with me. … … I can’t really. They ask why - for an explanation of it. I didn’t really experience it my first time. …… The culture here is to shake hands. If they are in front of other people I just, to respect them, I just do (JC24).*

Some of the students were happy to experience the differences and change practices while in an Irish environment. For others this would make them feel uncomfortable and they wished to maintain their own cultural practice regarding personal space whilst in Ireland, but sometimes felt that they were misunderstood.

*They don’t see why you cannot join a party even though you don’t drink. There are more things you have to consider. I can’t mix around, I can mix but not a lot of touching. We prefer not to mix (JC30).*

#### Inappropriate behaviour, attitudes and practices

Generally the students had not experienced discrimination in college. There were some instances described and in many cases students mentioned experiences of other students. These included not being treated well in restaurants; comments made or being shouted out on the street; bad experience of physical confrontation on the main city centre streets at night, and; snowballs being thrown at them.

*In Dublin there was one incident where a gentleman was rude. I was with my friends and they wore the Hijab. He wasn’t very nice. …… It was at a restaurant, he was a waiter. Just the way that he threw the menus, he wouldn’t explain it. He was rude when I asked anything about it, that sort of thing (JC22).*

However, what was interesting was that the above events were not viewed by respondents as serious. What was more upsetting for the students was when they experienced misunderstanding or discrimination from by their peers or people they felt were their friends.

*… there was this one night the other nationalities were going to a club and I decided to say no and they say, you’re boring, what are you going to do, stay home and study? It’s not that. I just don’t go to clubs (JC30).*

#### Dress

Students felt comfortable wearing the clothing of their choice or culture and were also comfortable with what other students wore. There were instances outside college, mentioned above, where comments had been made or shouted out by passers-by regarding their dress.

*During the Aidilfiltri*^1^*we used to wear something called Baju Kurung, but there is an Irish word for it. There is a skirt. We wear our pants and we wrap our waist with a short skirt and it’s not a skirt actually but when I pass through XXX Street there is an old guy say, nice skirt man. I don’t think it’s offended me but if that can be included as culturally inappropriate. (JC28)*

In many cases international students were relieved at the clothing worn by people in Ireland.

*I think just expecting their appearance, their clothes and costume. Maybe they are more open and sexy but it’s not. When I first come here it was cold so maybe they’re not sexy, just normal (JC15).*

### Phase 2

**Fig. 2:**
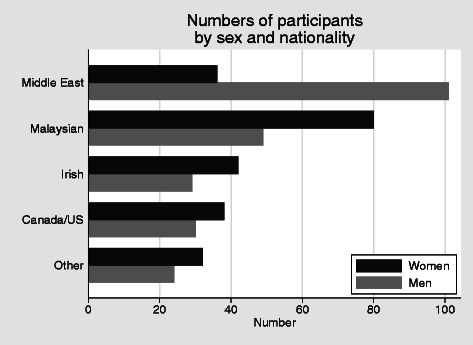
Numbers of participants by sex and nationality

There were 467 participants in the online survey. All but 69 (15 %) were from second medical year (Fig. 2). There is an almost 50/50 male: female ratio overall, but this conceals significant regional variation. Women outnumber men for all regions except the Middle East, where the 74 % of the students are men. The opposite ratio was found among Malaysian students, where only 38 % of the students were men (Fig. 2). These percentages are similar to those of the student body of the college as a whole.

### Participants

The initial Mokken scaling procedure was conducted with a scaling threshold set at *H* = 0.3. This produced three scales:

1. Positive experience of student life
2. Social alienation
3. Cultural alienation

This comprised negative items about feeling lonely, not fitting in, being homesick.

This included the items of being uncomfortable around cultural norms of dress and contact between the sexes.

We reran the Mokken procedure on the items in the first scale, with the threshold set to *H* = 0.4, in order to separate out correlated but potentially distinct subscales. This resulted in subscales of the positive experiences of student life scale:

Mokken scaling procedure identified the following subscales

- Social adjustment
- Careers support from friends
- Perceived perception of college
- Personal satisfaction with studying medicine

#### Positive experience of student life

**Fig. 3:**
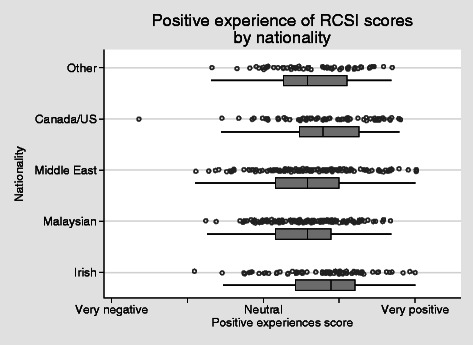
Positive experience of college scores by nationality. *H* = 0.353, apha = 0.89

It is worth noting that the majority of respondents had a positive experience of student life (Fig. 3). Levels were slightly lower in the Malaysian and Middle-Eastern students, and compared with the Irish these differences were significant (*P* = 0.005 and 0.041 respectively, using OLS regression). Adjusted for country of origin, there was no significant difference between the sexes.

**Fig. 4:**
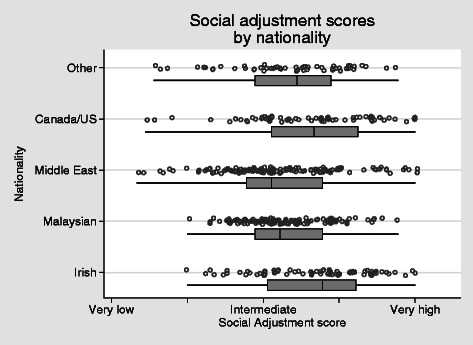
Social adjustment score by nationality *H* = 0.57, alpha = 0.90

Social adjustment scores ranged widely, though very few students extended into the lowest end of the range (Fig. 4). Taking Irish students as a benchmark, social adjustment was similar in Canadian/US students (*P* = 0.645) but was lower in those from Malaysia (*P* < 0.001), the Middle-East (*P* < 0.001) and other regions (*P* = 0.013).

**Fig. 5:**
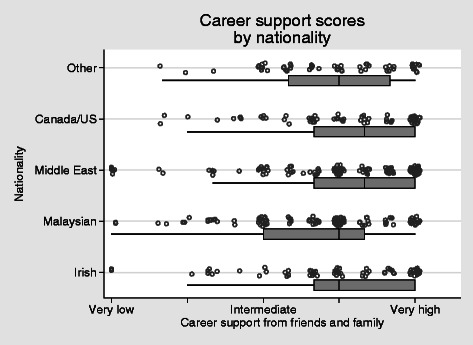
Career support from family and friends *H* = 0.64 alpha = 0.8

Career support from family and friends varied considerably within each student group (Fig. 5). None of the groups, however, differed significantly from the Irish students in their levels of support (OLS regression, robust standard errors).

**Fig. 6:**
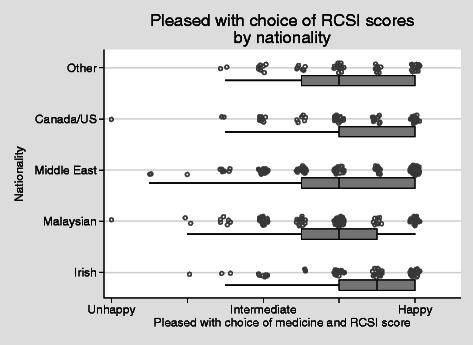
Happy with choice of college *H* = 0.68, alpha = 0.77

Most students were happy with their choice of college (Fig. 6). Two differences emerged in scores: men had lower scores than women (*P* = 0.003) and Malaysian students had lower scores than the Irish (as a baseline group, *P* = 0.001, OLS regression, robust standard errors). None of the other nationalities differed from the Irish.

#### Social alienation

**Fig. 7:**
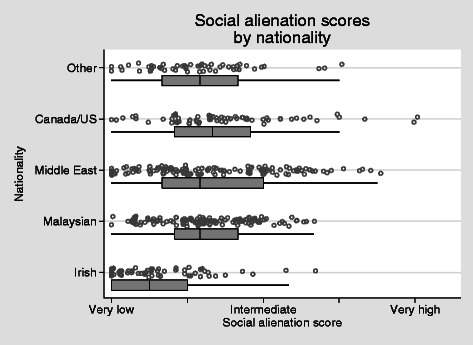
Social alienation scores by nationality *H* = 0.48, alpha = 0.82

The Irish students are a convenient benchmark for the social alienation scores (Fig. 7). Three quarters of Irish students fell into the very low category. By contrast, the scores of all other groups were very different to those of the Irish. In approximate terms, 75 % of students from other countries felt a level of social alienation that would be experienced by only the most alienated 25 % of Irish students. All nationality groups scored higher on social alienation than the Irish students (*P* < 0 · 001, OLS regression with robust standard errors). There was no difference in social alienation scores between the sexes adjusted for nationality (*P* = 0 · 51).

Alienation from western cultural values around gender and dress was predictably higher in Middle-eastern and Malaysian students, though even among these students there is considerable variation, with clusters of scores of zero on this scale (Fig. 7). However, in comparison with the Irish students as a benchmark, the difference is again marked, with three quarters of Irish students falling below the 25th percentile of scores for Malaysians or Middle-Eastern students.

#### Cultural alienation

**Fig. 8:**
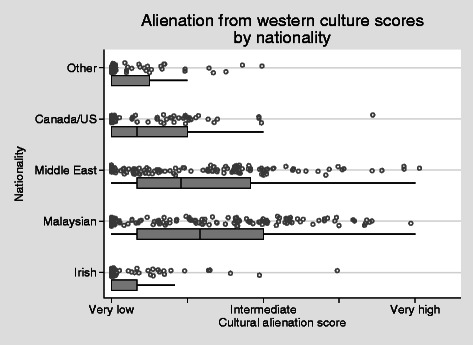
Alienation from western culture scores by nationality *H* = 0.48, alpha = 0.82

Alienation from western cultural values around gender and dress was predictably higher in Middle-Eastern and Malaysian students (both *P* < 0 · 001), though even among these students there was considerable variation, with clusters of scores of zero on this scale (Fig. 8). However, in comparison with the Irish students as a benchmark, the difference is again marked, with three quarters of Irish students falling below the 25th percentile of scores for Malaysians or Middle-Eastern students. Likewise, scores were higher in students from Canada/US (*P* = 0 · 014, Poisson regression with robust standard errors).

## Discussion

As transformative institutions work to ensure appropriate supports, both academic and pastoral, for international students, it is essential that we understand the challenges. These will differ depending on what part of the world the host country is located. This is confirmed when looking at students’ positive experience of the college and the interviewees’ comments around the positive environment and lack of discrimination. However, it is important to recognise the potential bias in the study whereby students may have wished to please academic staff implementing the research although measures were taken to address this. It is also worth noting the reported openness and friendliness of the Irish population in welcoming international students. Though there is a lack of formal research, media reports describe Ireland as being among the European countries most open to Muslims (http://theatln.tc/1AQURvH). Our findings are in keeping with a component of the Interaction Acculturation Model (IAM) [24] where the influence of host country attitudes is emphasised in the acculturation process. However, there is unfortunately an element of discrimination described by the students in the interview accounts above that, although not widespread is of concern from a national policy perspective. These accounts are similar to challenges described by international students in other host countries [11].

With regards to social adjustment it is clear from the interviews that some students were finding this more difficult than others. The discussion around friendships and greater ease in building relationships amongst international students as opposed to host students is in keeping with Dunne’s findings [15]. The students themselves see this as being one of the most important factors to help settling in, confirming other research findings [1, 3, 10]. Homesickness is described as a factor of concern for some whilst not for others – the latter being those who are used to living away from home. Again this resonates with some of the acculturation models described above where the same factors can be causes of stress to some and be benign to others [25] or considered to be minor and manageable stresses to many [26].

The results in the social adjustment scale (Fig. 4) range widely with the majority of students in the very high to intermediate scores. It is of note that the students from Canada and the United States are within a similar range to those from Ireland. Those from Malaysia and Middle East are significantly less well adjusted. These broad results are in keeping with Hofstede’s outline of cultural distance [27] which potentially offers some explanation. However, on closer examination of Fig. 4, it is clear that there is a wide distribution of results within regional groups. This again alludes to the diversity of individual responses to stressors and/or hassles and individual experiences and abilities to interact with others/adjust to the host environment, and argues that an understanding of cultural adaptation cannot be based on broad-brush approaches. Clearly, there are significant numbers of students adapting successfully. A more in-depth examination of the strategies used by these students is warranted.

Adaptation to cultural norms of the host society or perception of acceptance was explored in both phases. Students generally described feeling comfortable wearing their clothes of choice within college, which is not surprising given the numeric dominance of non-European cultures. They do outline some challenges experienced when the students circulate outside of the College environment. However, freedom of expression and friendliness of the host culture are generally described positively.

With regards to cultural practice and personal space, students described clear challenges with regards to proximity to the opposite sex, hand shaking and hugging. These are areas of concern to some individuals. While others were open to adapting to the host country norms others were very uncomfortable to do so and describe feeling misunderstood. When looking at alienation from western culture scores by nationality in Fig. 7, it is understood that cultural values around gender and dress are higher amongst those where the cultural distance is greater, i.e., Middle East and Malaysia, when compared to those students from the host country. It is clear that the majority of international students (75 %) fall below the 25th percentile of scores for Malaysians or Middle Eastern students. Interestingly this is also the case for those students from Canada and the United States where cultural distance could be understood to be similar. There is also a spread of results within the groups, suggesting that factors other than just region of origin; perhaps previous travel as a result of globalisation is playing a role in this case or indicating individual reaction to stressors and hassles.

One also needs to consider how important it is for students to fully acculturate given that they will be expected to return to their home countries post-graduation to practice medicine, which represents the sojourner nature of the international student. Given that this study was conducted on students in the first and second year of a five year programme it would be very interesting to track them longitudinally to assess if the average scores on the cultural and social alienation scales within cultural/regional cohorts reduce over time as the students adapt to the host culture.

The peer led interviews took place 3 months into the programme. The online survey was run twelve months post the interviews. Hence, first year students were being evaluated at the same time period one year later. We do of course appreciate that the qualitative data and the online survey capture student opinion and information at one particular time point and does not capture the dynamics of the acculturation process as the student progresses through the programme. Research indicates that students from different cultural backgrounds can initially mix well. However, over a period of time there was a decline in social interaction between cultures. Friendships and social learning were based on the same cultural background [28, 29]. Hence social adjustment is more of a dynamic than a static phenomenon. This will be particularly the case with this cohort of students as the learning environment will move from a predominantly “classroom” educational environment toward an increasingly ‘apprentice’ healthcare environment over time.

It will be interesting to follow this group over time in the clinical environment to learn if by continually mixing the students’ study groups as is normal practice within this institution, there is an influence on social adjustment or if the results will be in line with previous research findings. Furthermore previous research has demonstrated that when there are large cohorts of students, they tend to make up similar home culture groups. Those students with smaller numbers of co-nationals in the class are however forced to interact more with other cultures - hence potentially increasing levels of adjustment to the host country and they interact more with host students [30]. One theme that arose from the interviews was a strong sense that having large numbers from one international region could act as a barrier to integration – this is supported by previous research findings [29].

The phase 2 analysis demonstrates a clear clustering of students based on nationality though still illustrating a wide distribution within these groups when using the Mokken scales. This supports the argument that there are factors other than nationality influencing students’ adjustment to the host society and institution. Some students from countries where cultural distance is considered to be great, e.g., Middle Eastern countries and Malaysia were seen to be as well adjusted to western culture as the students from the host country. There is a need to understand clearly what other factors are involved in determining this. Indeed, this could be a very useful tool for institutions taking international students in identifying those that will adjust well the new environment and those that may be challenged in doing so. Further analysis of the data and research studies are warranted in order to decipher any particular characteristics that aid such adaptation for students.

It is important to point out that for the majority of students the experience in the host institution is perceived by them to be a positive one. Much of the literature discussed the challenges involved in acculturation as opposed to concentrating on the very positive aspect of having an opportunity to travel and learn about other cultures while studying at higher level. The overall findings of this study demonstrate that there are very few students with great difficulties However, a non- response rate of circa 50 % could mean that those students who did not respond to the on-line survey differed in some systematic way to those who did respond. In this the non-responders might have scored higher or lower on the measured scales. The reported findings, demonstrate good adjustment generally. This would be expected given that many of these students are government sponsored students and thus must be competitively among the most academically able and ambitious in their countries. Hence, they have demonstrated strength of character and focus to have been selected to attend a medical school outside of their own country.

## Conclusions

Overall student adjustment to a western third level college was good. Students from regions where cultural distance is greatest reported more difficulties. It is noteworthy that students from these regions also demonstrate very good adaptation, while some from Ireland and more similar cultural backgrounds were also struggling. It is clear that acculturation is more complex than being associated with cultural distance and worth further exploration.

The responsibility of a third level institution aiming to admit international students is to have the appropriate supports in place to assist those who find challenges and to assist them to manage these in a constructive manner, without expecting full acculturation or assimilation. This again is the mark of a transformative institution prepared to invest in education and supports while maximising the potential to accommodate international students.

This study signals changes to be made to our College Induction Programme, emphasising to students that it is not unexpected for some to have real problems adapting and that they should not be uneasy about coming for assistance. These messages will be replicated in the Personal Tutor Induction Programme and with student welfare officers so staff are primed to ask individual students questions referring to acculturation, e.g., how are they settling in; if there are any particular challenges that they are experiencing; are they making friends with students from their own background and also students from other cultures or countries. The experience here is likely to be similar in some respects in other international institutions - and of value as institutions reflect on the experiences of international students. Modelling good practices in assisting international students to settle into a new environment is an important part of a wider international diplomacy which provides messages for the host institution and region as much as for the incoming students and their host nations. Striving to get it right is thus part of the broad mutual benefit that international education can bring to the wider society in which these young people will live and work and shape the next generations.

## Footnotes

1 The Festival of the Breaking of the Fast, called Hari Raya Aidilfitri, is a religious holiday celebrated by Muslims. Hari Raya Aidilfitri is the day that marks the end of Ramadan, the Islamic holy month of dawn-to-sunset fasting.
